# The overlooked trio: sleep duration, sampling time and physical exercise alter levels of olink-assessed blood biomarkers of cardiovascular risk

**DOI:** 10.1186/s40364-025-00776-0

**Published:** 2025-04-29

**Authors:** Luiz Eduardo Mateus Brandão, Lei Zhang, Anastasia Grip, Mun-Gwan Hong, Emil Kåks, Rui Benfeitas, Fjola Sigurdardottir, Kaj Blennow, Henrik Zetterberg, Daniel Espes, Torbjørn Omland, Payam Emami Khoonsari, Christian Benedict, Jonathan Cedernaes

**Affiliations:** 1 Department of Medical Sciences, Uppsala University, Uppsala, Sweden; 2 Department of Medical Cell Biology, Uppsala University, Uppsala, Sweden; 3 SciLifeLab, Stockholm University, Stockholm, Sweden; 4 K.G. Jebsen Centre for Cardiac Biomarkers, University of Oslo, Oslo, Norway; 5 Clinical Neurochemistry Laboratory, Sahlgrenska University Hospital, Mölndal, Sweden; 6 Department of Psychiatry and Neurochemistry, Institute of Neuroscience and Physiology, Sahlgrenska Academy at the University of Gothenburg, Mölndal, Sweden; 7 Paris Brain Institute, ICM, Pitié-Salpêtrière Hospital, Sorbonne University, Paris, France; 8 Division of Life Sciences and Medicine, and Department of Neurology, Institute on Aging and Brain Disorders, Neurodegenerative Disorder Research Center, University of Science and Technology of China and First Affiliated Hospital of USTC, Hefei, People’s Republic of China; 9 Department of Neurodegenerative Disease, UCL Institute of Neurology, London, UK; 10 Dementia Research Institute at UCL, London, UK; 11 Kong Center for Neurodegenerative Diseases, Clear Water Bay, Hong Kong, China; 12 Wisconsin Alzheimer’s Disease Research Center, School of Medicine and Public Health, University of Wisconsin, University of Wisconsin-Madison, Madison, WI USA; 13 SciLife Lab, Uppsala University, Uppsala, Sweden; 14 Department of Pharmaceutical Biosciences, Uppsala University, Uppsala, Sweden

**Keywords:** Aerobic Exercise, Cardiometabolic Risk Factors, Cardiovascular Disease Risk, Inflammation, Sleep Curtailment

## Abstract

Biomarker profiling from biofluids such as blood are widely measured in clinical research, using for example Olink proteomics panels. One such research focus area is cardiovascular disease (CVD), for which chronic sleep restriction (SR) is a risk factor. However, it remains unclear whether blood levels of commonly measured CVD biomarkers are sensitive to acute dynamic factors such as SR, physical exercise (PEx), and time of day. In this crossover design, 16 normal-weight, healthy men underwent three highly standardized in-lab nights of SR (4.25 h/night) and normal sleep (NS, 8.5 h/night) in randomized order, with 88 CVD blood protein biomarkers quantified using the Olink technology (and selected validation using ELISA) in the morning, evening, and immediately before and repeatedly after 30 min of high-intensity exercise. We found significant time-of-day-dependent changes in several CVD biomarkers. Whereas several proteins were exercise-induced across sleep conditions (such as the canonical exerkines IL- 6 and BDNF), exercise-induced proteomic dynamics differed in response to recurrent SR, compared with following NS. Moreover, SR compared with NS resulted in a biomarker profile previously associated with increased prospective risk of several CVDs across large-scale cohorts (such as higher circulating levels of IL-27 and LGALS9). Our findings highlight how dynamic physiology can modulate CVD biomarker levels. These results also underscore the need to consider sleep duration as a key determinant of cardiovascular health—an emphasis reflected in recent American Heart Association guidelines. Further studies in women, older individuals, and patients with prior CVD, and across different chronotypes and dietary schedules are warranted.

**Supplementary Information:**

The online version contains supplementary material available at 10.1186/s40364-025-00776-0.

## Main text – correspondence submission

To the Editor,

Sleep disruption, including chronic sleep restriction (SR), has been associated with an increased risk of cardiovascular disease (CVD) and cardiovascular mortality [1, 2]. Healthy sleep duration was also added to the American Heart Association’s 2022 recommendation for cardiovascular health assessments [3]. Prospective studies often use proteomics to identify biomarkers that are associated with the risk of e.g., CVDs [4, 5]. However, despite SR being widespread [6, 7], such studies often fail to directly establish or take into the potential impact of recent sleep duration, or other physiological dynamic parameters, such as sample time and physical exercise (PEx). Notably, PEx may counteract some of the adverse cardiometabolic effects of poor sleep – however, without fully offsetting its adverse impact on CVD mortality [2, 8, 9]. This highlights the complex interplay between sleep, exercise, and cardiovascular health, which helped motivate our present proteomics-focused investigation. Using highly standardized in-lab conditions, our primary aim was to measure how circulating levels of a range of proteins implicated in promoting cardiovascular health or CVD [4, 5, 10], are acutely impacted by SR versus normal sleep (NS), as well as by concurrent morning-to-evening dynamics and in response to acute PEx.

### SR- and time-of-day-dependent dynamics in blood proteomics

**Fig. 1:**
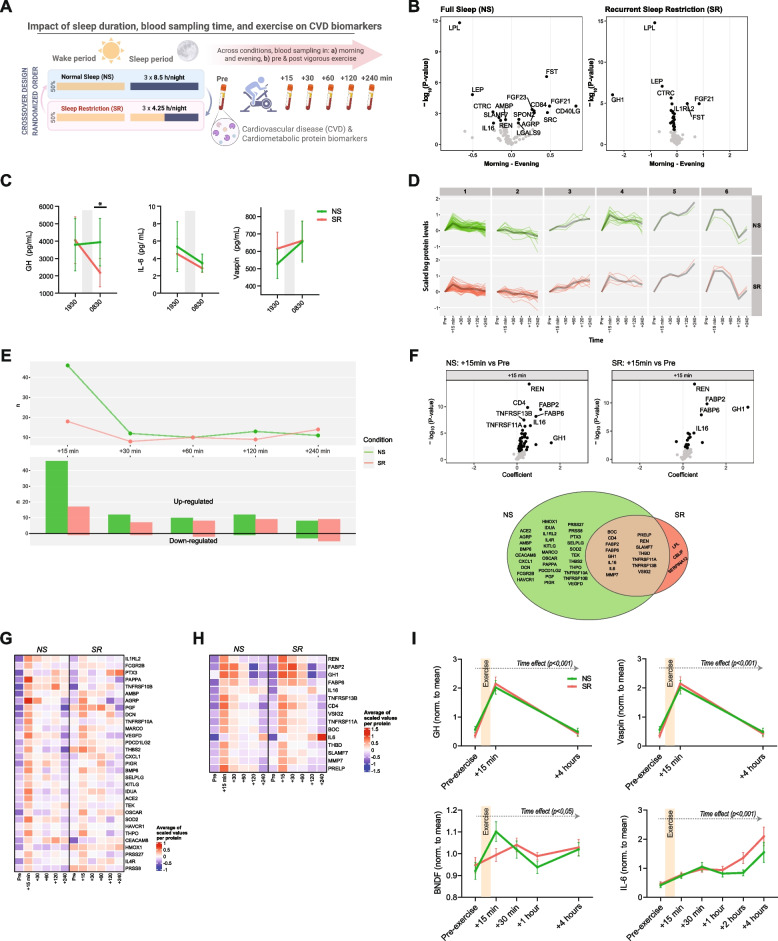
(below). Sleep restriction, time of day and physical exercise dynamically modulate proteomic CVD biomarker levels (related to Fig S1 - 2). **A** Overview of the study protocol, illustrating the randomized crossover 2-session study design. **B** Volcano plot showing proteins that exhibited significant changes from morning to evening in the normal sleep (NS) and recurrent sleep restriction (SR) conditions. A positive coefficient indicates higher protein levels at the given post-exercise timepoint. Note that the y axis shows the uncorrected *p* values; significant proteins (FDR-corrected *P*<0.05) are shown in black. **C** Protein quantification based on ELISA for evening-to-morning dynamics of GH, IL- 6 and VASPIN, analyzed by repeated measures ANOVA, across the two sleep conditions. * indicates P < 0.05, for post-hoc analysis at 0830 h (Šídák's multiple comparisons test). **D** Cluster analysis for the exercise timepoints. Shows line graphs for each cluster, in the NS (green, top) and SR (red, bottom) conditions. Proteins that appear in each cluster are plotted within the cluster as a single line (mean value) across the acute exercise timepoints (Pre-exercise to + 240 min). See also figure S2 A. **E** Line graph showing the number of proteins that exhibited significant acute exercise effects in the NS (green) and SR (red) conditions, with each timepoint being compared with protein levels at the pre-exercise timepoint. The lower graph shows the directionality (up- or downregulated) of the changes at each timepoint. **F** *Top*: Volcano plots showing proteins that exhibited significant acute exercise effects (+ 15 min vs. Pre-exercise) in the NS (left) and SR (right panel) conditions. A positive coefficient indicates higher protein levels at the given post-exercise timepoint. Note that the Y axis shows uncorrected *p*-values; significant proteins (FDR-corrected *p *<0.05) are shown in black. *Bottom*: Venn diagram shows proteins that changed uniquely within each condition, and those that were shared between the NS and SR conditions. **G** Heatmap showing acute exercise effects (+15 min vs. Pre-exercise) that were only significant in the NS condition. **H** Heatmap showing significant acute (+ 15 min vs. Pre-Exercise) exercise effects observed across both sleep conditions. **I** ELISA validation of Growth hormone (GH), visceral adipose tissue-derived serpin (VASPIN), Brain-derived neurotrophic factor (BDNF) and interleukin 6 (IL- 6). Values are normalized within each subject, to the mean of each individual’s values. Analyzed by repeated measures ANOVA, across the two sleep conditions. All analyses based on *n* = 16 within-subject analyses

To probe CVD biomarker dynamics, we used a randomized within-subject design: 16 normal-weight men underwent two conditions across three consecutive in-lab nights: a) NS (8.5 h/night), and b) recurrent SR (4.25 h/night; see Supplemental Methods). Across both conditions, 88 CVD protein biomarkers were quantified using the Olink CVDII panel, from serum drawn in the morning and evening, and before and repeatedly after high-intensity PEx (Fig. 1A).

First focusing on time-of-day-dependent dynamics, we found that a subset of proteins, such as leptin and lipoprotein lipase (LPL), exhibited significant morning-to-evening changes across conditions (Table S1 A-B). A greater proportion of the proteins exhibited significant morning-to-evening dynamics during SR (33%, FDR < 5%) compared with NS (18%), as also indicated by ELISA validation (Fig. 1B-C). In contrast, significant early morning dynamics (pre-PEx, ~0830 h to ~1030 h) were only evident after NS (40% decreased) (Fig. S1).

### Physical exercise modulates CVD biomarker levels

To next assess how biomarker dynamics are impacted by acute PEx, we had participants undergo 30 min of high-intensity cycling on the third day of each in-lab sleep condition. SR compared with NS resulted in an altered proteomic response (hierarchical clustering-based *P* = 0.048, Figs. 1D and S2 A). Indeed, immediately post- vs. pre-PEx (~ 15 min after the offset of PEx vs. ~ 20 min before PEx), blood levels of 46 proteins significantly increased in NS, compared with only 18 proteins under SR (Fig. 1E-H and S2 C; Table S2). Among these, proteins were exclusively higher in the NS condition, and three proteins were significantly altered only in the SR condition (two increased, one decreased; Fig. 1F). Sleep condition-specific differences following PEx may in part have been driven by slightly higher pre-PEx levels following SR vs. NS (Fig. S2B). Nevertheless, across both sleep conditions, 15 proteins increased in blood immediately post-PEx (Fig. 1H). This comprised several proteins implicated in the beneficial effects of PEx, including the canonical exerkines IL- 6 and BDNF (Figs. 1I and S2D). In terms of magnitude, changes in immediate post-PEx-induced (vs. pre-Ex) protein levels were in general larger than the morning-to-evening dynamics: ~1.4-fold larger on a per-protein bases in the largest (n = 60) cluster (Fig. S3). During later post- vs. pre- PEx timepoints, significant increases and decreases in protein levels were similar across both sleep conditions (Figs. 1E and Fig S2E-F; Table S3 A-D).

### SR promotes a biomarker profile associated with prospective CVD risk

**Fig. 2:**
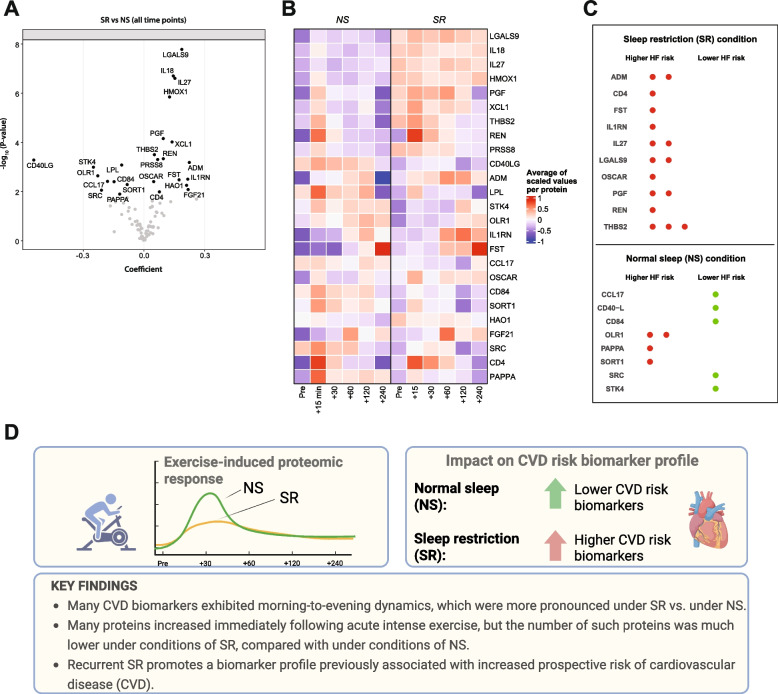
(below). Recurrent sleep restriction promotes changes in proteomic levels in a direction that have previously been associated with prospective risk of cardiovascular disease. **A** Volcano plot comparing relative proteomic levels after three nights of full sleep (NS) with three nights of sleep restriction (SR), using a mixed effects model. The X axis represents model coefficient value, where values above 0 indicate higher values in SR compared with NS condition. Note that the Y axis shows uncorrected *p*-values; significant proteins (FDR-corrected *P* < 0.05) are shown in black. See also Table S4 A. **B** Heatmap that shows relative levels for proteins that were significantly different (as seen in panel A) after three nights of NS compared with three nights of SR. The columns show levels across the pre- and post-exercise timepoints. **C** Overlap of proteins with significantly altered levels after three nights of SR compared with NS in our study, with those identified as significantly predictive of greater (red circles, left column) or lower (green, right column) risk of heart failure (HF), across the 3 cohorts analyzed in [4] (see also Table S4B). The number of circles per row indicate across how many of the 3 studied prospective cohorts that the association with HF was identified as being significant. Proteins have been sorted alphabetically. All analyses based on *n *= 16 within-subject analyses. **D** Summary of the present study findings

Finally, given the association of chronic SR with CVDs, we wanted to investigate how SR per se may alter circulating levels of CVD biomarkers. Notably, we found that regardless of PEx – i.e., across timepoints comparing SR with NS – recurrent SR resulted in 16 proteins with significantly higher levels, and 9 with significantly lower levels. The upregulated set included several stress, interleukin, and chemokine-related proteins (Fig. 2A-B, Table S4 A). By comparing the changes in levels of the Olink-derived biomarkers used in our study with associations for these Olink biomarkers in large prospective CVD cohorts (largest n = 44,313) [4, 5], we found that SR vs. NS resulted in a biomarker profile associated with a higher risk of heart failure, coronary artery disease, and atrial fibrillation (Fisher’s exact test *P* = 0.006 for data in Fig. 2C; *P* = 0.003 for data in Table S4B). In contrast, NS vs. SR predominantly increased proteins linked to a lower CVD risk (Fig. 2C).

### Summary and future directions

Our findings (Fig. 2D), based on highly standardized in-lab conditions, indicate that even short-term sleep restriction can produce a biomarker profile associated with increased CVD risk. This aligns with recent American Heart Association guidelines [3]. For enhanced precision medicine, recent sleep and exercise history, and the timing of also blood samples and meals, should be considered when evaluating proteomic markers for predicting cardiovascular health. Further studies in women, older individuals, different chronotypes, and patients with CVD are warranted.

## Supplementary Information

Supplementary Material 1.

Supplementary Material 2.

## Data Availability

Anonymized data underlying this article will be shared by the corresponding author on reasonable request to qualified researchers from accredited academic institutions.

## Abbreviations

CVD: Cardiovascular disease
FDR: False discovery rate
NS: Normal sleep
PEx: Physical exercise
SR: Sleep restriction
