# Supplementary material for: The overlooked trio: sleep duration, sampling time and physical exercise alter levels of olink-assessed blood biomarkers of cardiovascular risk
Source: Biomark Res. 2025 Apr 29;13:67. doi: 10.1186/s40364-025-00776-0 (PMC12038921; doi:10.1186/s40364-025-00776-0)
Supplement: Supplementary file 1 — Supplementary Material 1. [file 40364_2025_776_MOESM1_ESM.pdf]

Supplemental material:

- **Figure S1 (page Sp.2).** Changes across early morning timepoints in levels of CVD biomarker proteins (related to **Fig. 1**)
- **Figure S2 (page Sp.3).** Exercise-induced proteomic dynamics under conditions of normal sleep (NS) and recurrent sleep restriction (SR) (related to **Fig. 1**)
- **Figure S3 (page Sp.4)** Evening-to-morning dynamics in CVD biomarker levels are similar in magnitude and in the same or opposite direction to those induced immediately after acute exercise
- **Supplementary methods (pages Sp.5-6).** Expanded methods section, including more details about the clinical study.
- **Supplementary references (page Sp.7)**
- Separate Excel file:
  - For **supplementary tables S1-S4**.

Figure S1. (below)

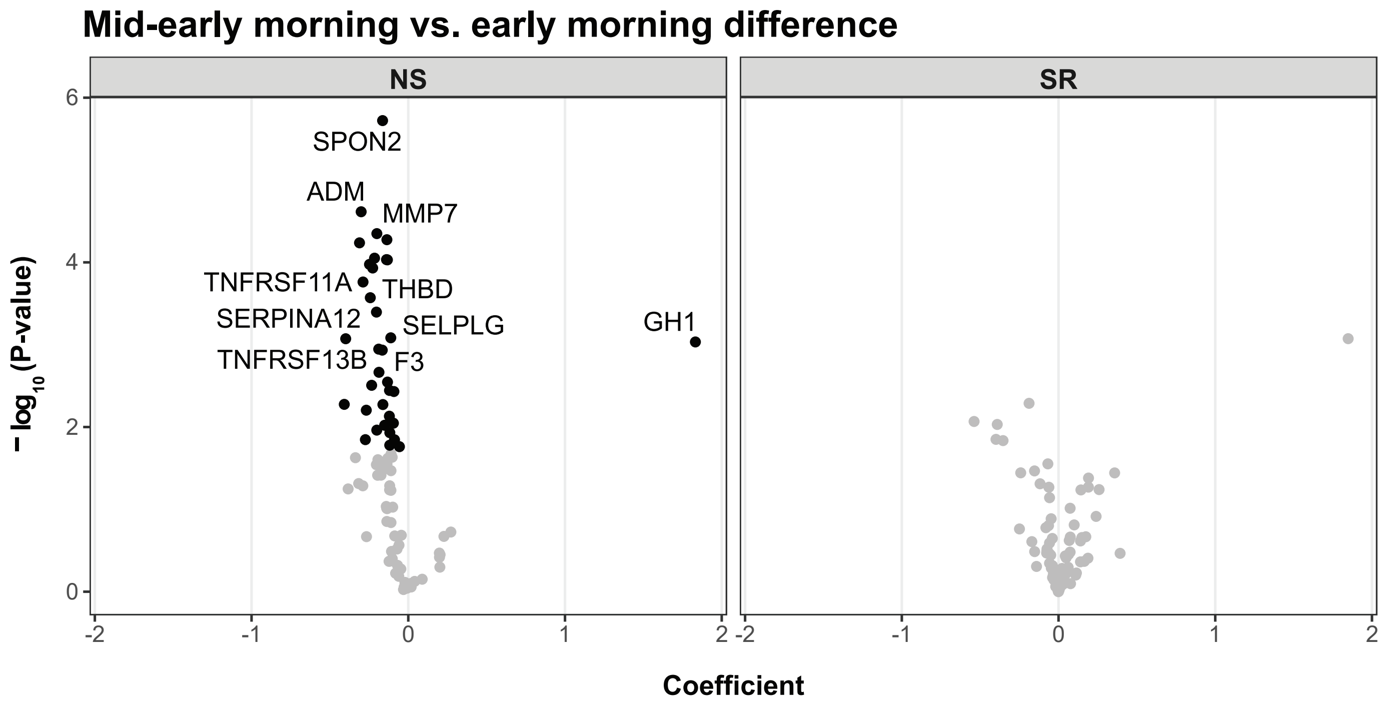

Figure S1. Changes across early morning timepoints in levels of CVD biomarker proteins (related to Fig. 1). Volcano plots for the comparison between the two fasted morning (pre-exercise) timepoints after three in-lab nights, in the normal sleep (NS) and recurrent sleep restriction (SR) conditions. For the model contrast in the mixed modeling, we used the later (~1030h) compared with the earlier timepoint (~0830h), meaning that negative coefficients indicate higher relative protein values at the earlier morning timepoint. The Y axis shows the uncorrected p value; significant proteins are represented in black dots (FDR-corrected for multiple comparisons); non-significant protein changes are shown as grey dots. All analyses based on n=16 within-subject analyses.

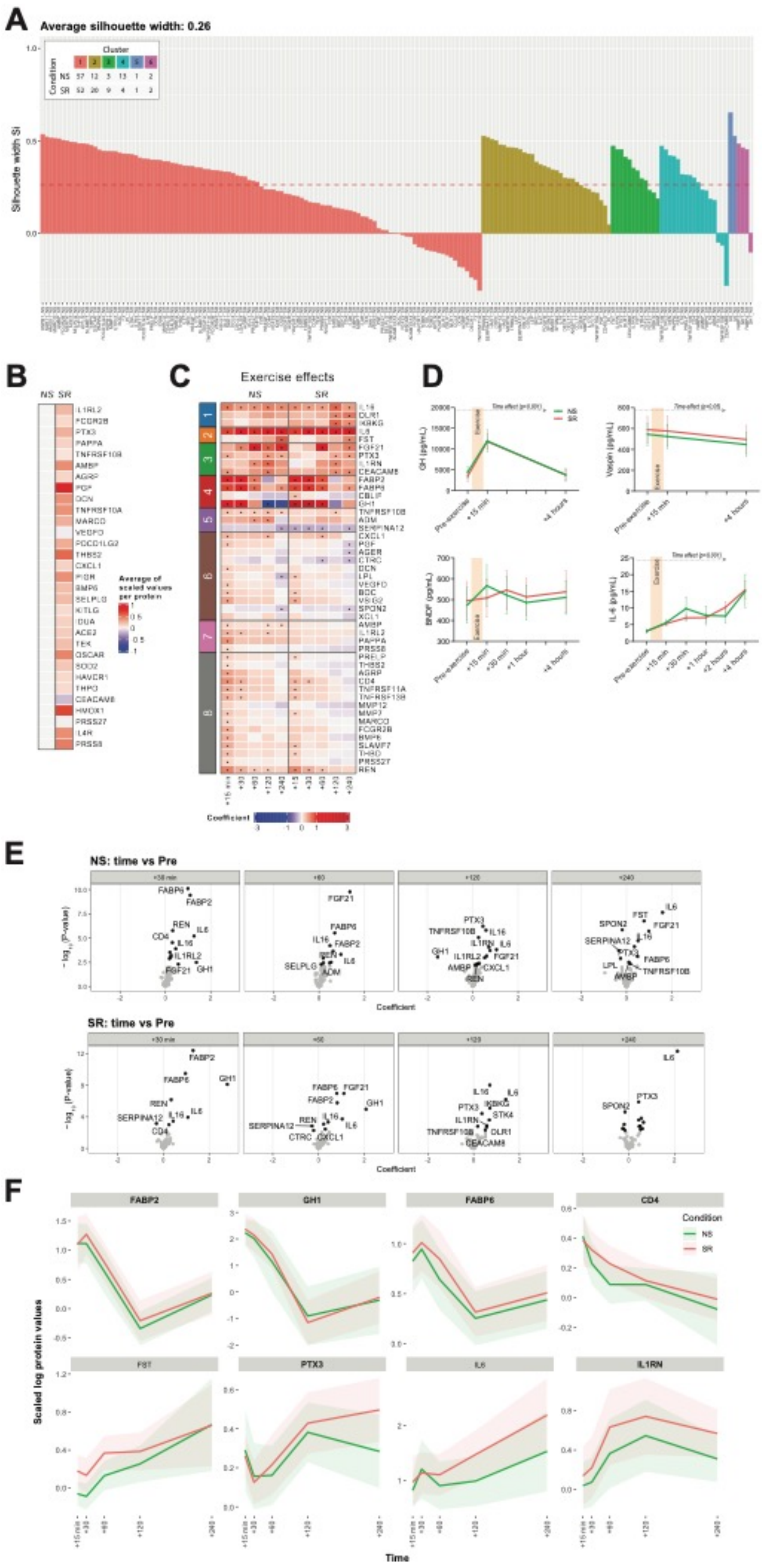

Figure S2 (left)

Exercise-induced proteomic dynamics under conditions of normal sleep (NS) and sleep restriction (SR) (related to Fig. 1).

A) Silhouette graph for the cluster analysis for the exercise-specific proteomic dynamics (see also Fig 1D). Table to the right shows the number of proteins assigned to each cluster, across the two sleep conditions.

B) Heatmap that shows average pre-exercise protein levels for proteins that were later significantly altered by acute exercise (+15 minutes versus pre) only in the NS condition. For each protein, the average levels have been set to 0 for the NS condition.

C) Heatmap that shows proteins with significant overall time effects in response to exercise, from the mixed effect model ANOVA analysis. Proteins have been grouped based on the similarity of the coefficients of the mixed effect model. Each dot in the heatmap indicates that the protein exhibited a significant time effect compared with the immediate pre-exercise morning timepoint ($P<0.05$, FDR-corrected for multiple comparisons).

D) ELISA validation of Growth hormone (GH), VASPIN, Brain-derived neurotrophic factor (BDNF) and interleukin 6 (IL-6). Shows absolute protein values.

E) Volcano plots for the protein changes in response to exercise, at +30, +60, +120, and +240 minutes post-exercise, with all comparisons done against the immediate pre-exercise timepoint, using mixed effects modeling (X axis represents model coefficient value, Y axis shows $-\log_{10}$(P value)). Positive coefficients indicate higher protein levels at the given post-exercise timepoint. The Y axis shows the uncorrected p value; significant proteins (FDR-corrected) are represented by black dots and by name wherever possible. See also **Table S3**. All analyses based on n=16 within-subject analyses.

F) Expression profiles of individual proteins from the Olink data, for the third day across the exercise timepoints (from pre-exercise to +240 min post exercise).

Figure S3. (below)

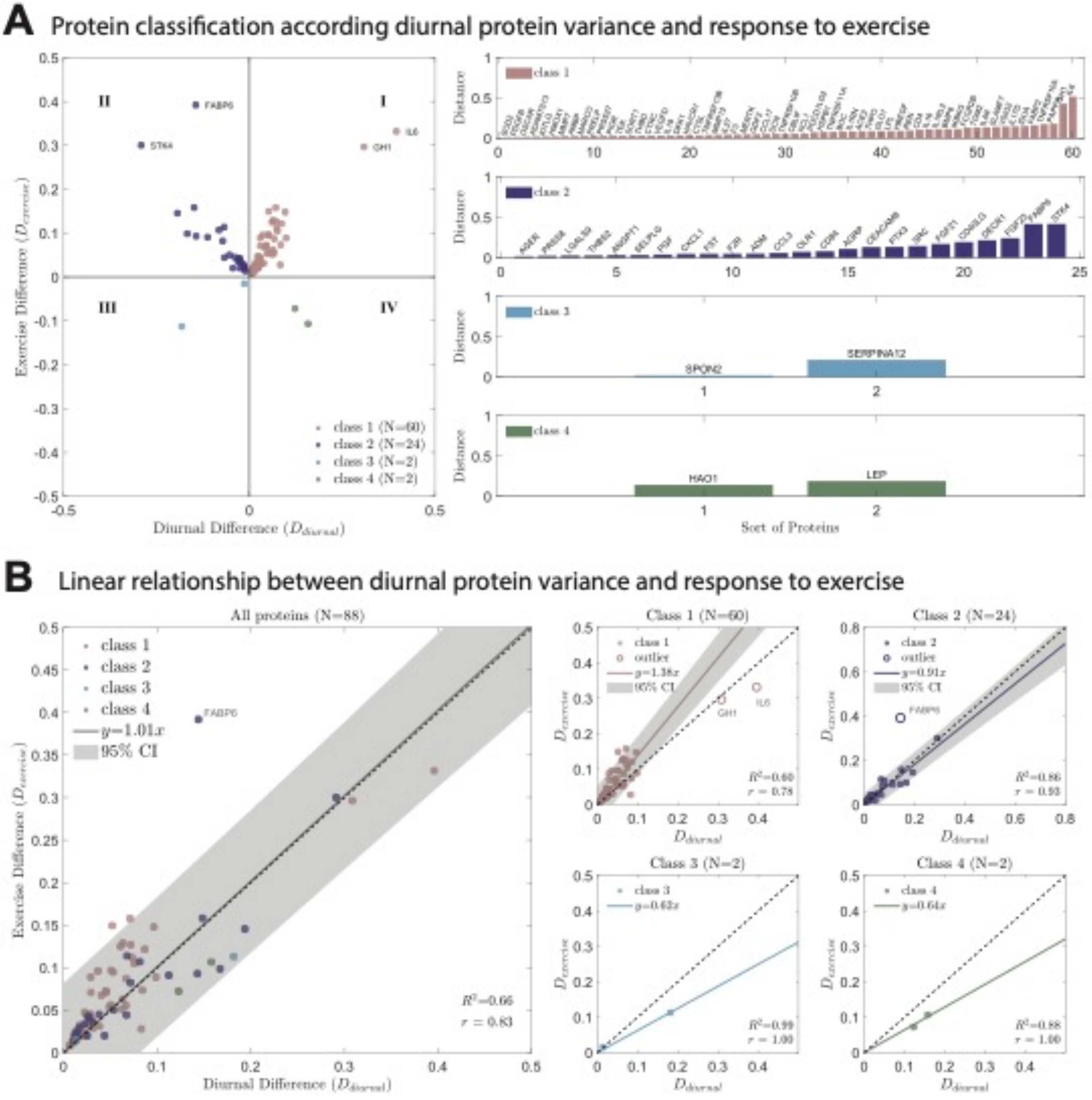

Figure S3: Evening-to-morning dynamics in CVD biomarker levels are similar in magnitude and in the same or opposite direction to those induced immediately after acute exercise.
Classification and comparison of magnitude of morning-to-evening & immediate acute exercise responses, with comparisons done within each subject, using the average across the two morning and one intervening evening timepoints (for the morning-to-evening analysis), and for the timepoint right after (+15 min) versus right before the bout of high intensity exercise, using values from both sleep conditions. Shows how proteins cluster in terms of **A)** their distance between the two types of comparisons (morning-to-evening ($D_{diurnal}$) and acute exercise response ($D_{exercise}$); see methods for more details), and **B)** the linear relationship and correlation coefficients for the comparison of these two physiological dynamics, for each individual protein cluster.

Supplemental methods

Recruitment and participant information

The present study has been described previously (1). In total, 16 healthy and normal-weight Caucasian men (age 23 ± 0.7 years; BMI 23 ± 0.5 kg/m^2), without any chronic neurological, psychiatric or somatic health conditions, were screened and enrolled by J.C., and successfully underwent both study conditions.

All participants reported consumption below 5 units of alcohol per week; none had engaged in shift work within the past six months; nor had participants traveled across >1 time zone in the four weeks prior to the study (and no such travel was allowed in between the study sessions). Participants also underwent strict screening for cardiovascular health, and none had any history of cardiovascular disease (general heart conditions, hypertension, hypotension, rhythm disorders); none had any first-degree family history of cardiovascular disease (general heart conditions; rhythm disorders); one participant reported a first-degree relative with known hypertension, but this individual had a normal resting blood pressure (systolic blood pressure (SBP) < 130; diastolic blood pressure (DBP) <80 mm Hg). All participants had normal resting blood pressure (SBP >90 but <130 mm Hg, and DBP >60 but <90 mm Hg). We also verified by resting ECG that all participants had normal sinus rhythm (including normal QRS complex). Participants were also screened for normal fasting glucose levels (<6.0 mmol/L) and a normal oral glucose tolerance test (2-hour glucose values <7.8 mmol/L), as well as normal total leukocyte counts (<9 × 10⁹/L). Plasma creatinine (<105 μmol/L), total cholesterol (<6.1 mmol/L), and triglyceride (<2.6 mmol/L) levels were also within the normal reference ranges established by the local hospital laboratory (Clinical chemistry, Uppsala University hospital).

The sample size was not based on any power calculation but was selected based on previous interventional studies that have examined the cardiometabolic effects of sleep restriction (SR) or acute exercise on healthy individuals (2-7). The study was carried out at the biomedical center at Uppsala University, during October 2014 to March 2015.

Using a crossover design with a randomized order of study conditions, the participants underwent both **a)** three nights of SR (4.25 h sleep opportunity per night, 0245-0700h) and **b)** three nights of NS (8.5 h sleep opportunity per night, 2230-0700h). In the SR condition, light exposure was kept below 5 lux at eye level during the extended wakefulness period (2230h-0245h). Sleep data for the study has previously been reported (1). For the daily in-lab meals (isocaloric breakfast, lunch and dinner), energy requirements were individually calculated for each participant, using the Harris-Benedict equation, and were kept identical for each participant across both conditions.

After the three in-lab nights of either SR or NS, participants underwent a strenuous bout of exercise on a stationary bike. The 30-min exercise bout was preceded by a 5-min warmup, at 25% of the subsequent intensity. The 30-min workout was done at 75% of each participant's calculated $VO_2^{maxReserve}$ (calculated from a prior sub-maximal VO_2^{max} test on the same bike). Fasting blood was collected ~0830h on the 2nd and 3rd in-lab morning in each condition, as well as in a fasted state in the evening in between these two mornings (~1930h). After the morning blood sample of the 3rd day, blood was sampled before (~1030h) and repeatedly after the 30-min exercise bout; post-exercise at 15, 30, 60, 120 and 240 minutes after completion of the exercise bout. Plasma and serum were collected in PST II and SST II tubes (BD Sweden), respectively. Following centrifugation for 10 minutes at 4°C, samples were immediately aliquoted and frozen on dry ice. Apart from the bout of exercise, participants remained in a semi-recumbent position on the 3rd morning, until the blood sampling period was over.

The study was approved by the regional Ethics Committee in Uppsala, Sweden (Dnr 2014/242/1), conformed to the principles outlined in the Declaration of Helsinki, and was conducted following written and orally informed participant consent.

Proteomics panel

The CVDII immunoassay panel (Olink Bioscience, Uppsala, Sweden) was used to quantify the levels of 92 CVD-focused biomarkers from serum, as previously described (8). To reduce intra- and inter-plate assay differences, the order of samples was randomized across subjects, conditions and timepoints, by a statistician blinded to the study hypotheses. All plates were subsequently run in one batch.

ELISA validation

For validation and quantification of some of the key markers, we used commercial ELISA kits or clinical chemistry analyzers, to measure concentrations of BDNF (R&D Systems, Minneapolis, cat #DBNT00) GH (R&D Systems, Minneapolis, cat. #DY1067), IL-6 (Cobase 8000 e801), and Vaspin/SERPINA12 (Adipogen Life Sciences, Füllinsdorf, cat. #AG-45A-0017Y). Due to the far greater volume requirements, we used fewer post-exercise timepoints for several ELISAs, compared with the Olink assay.

Bioinformatics and statistical analyses

All data processing was done in R or Matlab. Given the repeated measures within-subject design, we utilized a linear mixed model, using the R packages lme4 (v1.1-32) and lmerTest (v3.1-3), with subject set as random factor and false discovery rate (FDR, Benjamini-Hochberg) set at 5%.

All serum samples passed Olink's internal QC validation, with an average 3% intra-plate CV, and an average 10% inter-plate CV. Normalized log_2-transformed output data (normalized protein expression (NPX) values) were generated by Olink Bioscience.

As the next step in quality control, we assessed the proportion of samples with NPX values below the protein-specific limit of detection (LOD). Proteins that had more than 15% of the total number of measured values assigned below the LOD, were removed from the analysis. This resulted in the proteins BNP (P16860), CA5A (P35218), ITGB1BP2 (Q9UKP3) and PARP1 (P09874) therefore being excluded. Of the remaining proteins, none had missing data.

Clustering based on Z-scaled NPX values was done using the R package NbClust (v3.0.1), using hierarchical clustering with Euclidean distance and complete linkage, and the optimal number of clusters determined using Rousseeuw's Silhouette method (9). The timepoints used comprised the pre-exercise to the +240 min post-exercise timepoint. Heatmap-specific clustering for the mixed model output was done using hierarchical clustering (squared root of doubled values of 1 minus Pearson's correlation coefficient set as the distance, with Ward's agglomeration method modified by Murtagh and Legendre). Visualization was done using the R package ggplot2 (v3.4.2).

To test whether proteins that were seen at significantly higher levels after a) SR or b) NS were significantly associated with proteins that have been prospectively associated with a **i)** lower or **ii)** higher risk of heart failure, we ran Fisher's test using the overlap with significant prospective CVDII protein markers (i.e., overlap with significant proteins with odds ratio <1 or odds ratio >1, respectively, presented in the adjusted model, i.e., Table 2, of Girerd et al. (8)). The cohorts investigated by Girerd et al. averaged around 2.7 to 8.1 years of follow-up, for a total of n=2315 participants. We also did a similar analysis for significant proteins in the study by Schuermans et al., for the conditions coronary artery disease (CAD), heart failure (HF) and atrial fibrillation (AF), over a median of 11.1 years in a cohort of 44,313 UK Biobank participants (10) (see **Table S4B**). Note that for Girerd et al, some proteins had been listed by different protein aliases, compared with the protein names for the Olink CVDII protein arrays that we also used.

To compare the dynamics on relative protein levels (NPX values) from morning-to-evening compared with the acute exercise-induced effect, we first calculated the relative deviation in protein values from the morning timepoints (average of the two circa-8-am morning timepoints) to the evening timepoint, and defined this as the "diurnal difference":

$$D_{diurnal} = \frac{|c_{evening} - c_{morning}|}{c_{morning}}$$

Similarly, the difference between protein levels at the timepoint right before exercise, to the timepoint 15 minutes after the bout of exercise was terminated (after vs. before), was used to calculate the "exercise difference":

$$D_{exercise} = \frac{|c_{after} - c_{before}|}{c_{before}}.$$

Based on these differences for all proteins, we classified the proteins into four groups, based on whether they increased or decreased for their diurnal difference ($D_{diurnal}$) versus exercise difference ($D_{exercise}$). Each group of proteins were then ranked in terms of their Euclidean distance in $D_{diurnal} - D_{exercise}$ space (**Figure S2A**). Furthermore, we used a linear model with a 95% confidence interval to fit all proteins, as well as the four groups of proteins, in the $D_{diurnal} - D_{exercise}$ space.

For ELISAs, values were analyzed using repeated measures 2-way ANOVAs (for sleep condition, timepoint and sleep*time interactions). Post-hoc comparisons were done using Šídák's multiple comparisons test. Datasets that were not normally distributed were first log-transformed. A significance threshold of 5% was used for all analyses.

Supplemental references

1. Martikainen T, Sigurdardottir F, Benedict C, Omland T, Cedernaes J. Effects of curtailed sleep on cardiac stress biomarkers following high-intensity exercise. ***Molecular metabolism***. 2022;58:101445.
2. Broussard JL, Ehrmann DA, Van Cauter E, Tasali E, Brady MJ. Impaired insulin signaling in human adipocytes after experimental sleep restriction: a randomized, crossover study. ***Ann Intern Med***. 2012;157(8):549-57.
3. Irwin M, Thompson J, Miller C, Gillin JC, Ziegler M. Effects of sleep and sleep deprivation on catecholamine and interleukin-2 levels in humans: clinical implications. ***J Clin Endocrinol Metab***. 1999;84(6):1979-85.
4. Dettoni JL, Consolim-Colombo FM, Drager LF, Rubira MC, Souza SB, Irigoyen MC, et al. Cardiovascular effects of partial sleep deprivation in healthy volunteers. ***J Appl Physiol (1985)***. 2012;113(2):232-6.
5. Fu F, Nie J, Tong TK. Serum cardiac troponin T in adolescent runners: effects of exercise intensity and duration. Int J Sports Med. 2009;30(3):168-72.
6. Middleton N, George K, Whyte G, Gaze D, Collinson P, Shave R. Cardiac troponin T release is stimulated by endurance exercise in healthy humans. ***J Am Coll Cardiol.*** 2008;52(22):1813-4.
7. Shave R, Ross P, Low D, George K, Gaze D. Cardiac troponin I is released following high-intensity short-duration exercise in healthy humans. Int J Cardiol. 2010;145(2):337-9.
8. Girerd N, Levy D, Duarte K, Ferreira JP, Ballantyne C, Collier T, et al. Protein Biomarkers of New-Onset Heart Failure: Insights From the Heart Omics and Ageing Cohort, the Atherosclerosis Risk in Communities Study, and the Framingham Heart Study. ***Circ Heart Fail***. 2023;16(5):e009694.
9. Lovmar L, Ahlford A, Jonsson M, Syvanen AC. Silhouette scores for assessment of SNP genotype clusters. ***BMC Genomics***. 2005;6:35.
10. Schuermans A, Pournamdari AB, Lee J, Bhukar R, Ganesh S, Darosa N, et al. Integrative proteomic analyses across common cardiac diseases yield mechanistic insights and enhanced prediction. ***Nat Cardiovasc Res***. 2024.
